# Comparison of the radiographic outcomes and total blood loss between pinless navigation and conventional method in minimally invasive total knee arthroplasty

**DOI:** 10.1186/s13018-023-03534-w

**Published:** 2023-03-28

**Authors:** Shih-Hsiang Yen, Po-Chun Lin, Jun-Wen Wang

**Affiliations:** Departmaent of Orthopaedic Surgery, Kaohsiung Chang Gung Memorial Hospital, College of Medicine, Chang Gung University, 123, Ta Pei Road, Niao Sung District, Kaohsiung City, Taiwan

**Keywords:** Navigation, Total knee arthroplasty, Blood loss

## Abstract

**Background:**

Computer-assisted surgical navigation has been used in total knee arthroplasty (TKA) procedures for years trying to the accuracy of prosthesis placement. We conducted this prospective randomized clinical trial to compare the accuracy of the radiographic parameters of the prosthesis, total blood loss (TBL), and related complications, between a new pinless navigation system (Stryker OrthoMap Express Knee Navigation) and conventional method in patients undergoing minimally invasive (MIS) TKA procedures.

**Patient and methods:**

A consecutive series of 100 patients underwent unilateral primary TKA were randomly assigned into two groups: navigation group and convention group. The radiographic parameters of the knee implant and the alignment of lower limb were measured at 3 months after surgery. TBL was calculated according to Nadler’s method. The duplex ultrasonography of both lower limbs was performed in all patients to detect the presence of deep-vein thrombosis (DVT).

**Results:**

Totally, 94 patients have completed the radiographic measures. Only the coronal femoral component angle in the navigation group (89.12° ± 1.83°) had significant differences from that in the convention group (90.09° ± 2.18°) (*p* = 0.022). There were no differences in the rate of outliers. The mean TBL in the navigation group was 841 ± 267 mL, which was similar to that in the convention group at 860 ± 266 mL (*p* = 0.721). Postoperative DVT risk did not differ between the two groups (2% vs. 0%, *p* = 0.315).

**Conclusion:**

This pinless navigation TKA showed a comparable acceptable alignment compared with conventional MIS-TKA. There were no differences regarding postoperative TBL between the two groups.

## Introduction

Total knee arthroplasty (TKA) has been a successful surgery to treat end-stage knee disease for decades. Minimal invasive surgery total knee arthroplasty (MIS-TKA) is an advanced procedure to minimize soft tissue damage, reduce postoperative pain and blood loss, shorten hospital stay and recover time [1, 2]. However, some concerns may be associated with MIS-TKA. Smaller surgical visual field may lead to inaccurate bone cut and prosthesis position. Significant malposition of prosthesis will cause linear wear and implant loosening and is associated with inferior long-term outcome [3, 4]. Therefore, MIS-TKA procedure requires a longer learning curve to achieve better outcomes [5].

Computer-assisted surgical (CAS) navigation has been used in TKA procedures for years trying to improve the accuracy of prosthesis placement and postoperative mechanical alignment. Some studies have been demonstrated that the computer navigation system used in MIS-TKA can decrease the number of outliers with more than 3° deviation from the mechanical axis [6–10]. Moreover, navigated TKA avoids the use of intramedullary guide and preserves the medullary cavity of femur, so the risks of bleeding and venous thromboembolism are reduced. However, traditional navigation system requires additional procedure to set reference arrays with pin fixation of femur and tibia. Pin wound complications including bleeding, infection, neurovascular injury and iatrogenic fracture were reported [11–14]. Therefore, controversies exist regarding traditional CAS in views of increased operation time, additional wounds in the femur and tibia, and related complications, and whether decreasing postoperative blood loss and venous thromboembolism (VTE) in CAS-TKA.

The new pinless navigation system for TKA procedures has been developed [15–17]. In the pinless navigation system, the navigation tools were fixed in the surgical field without complications that are associated with use of femoral or tibial reference tracker pins or intramedullary alignment guides. This advantage meets the rationale of MIS-TKA to take care of both minimally invasive procedures and accuracy of prosthesis placement. Meneghini et al. [18] demonstrated that the blood loss could be reduced by using this new pinless navigation system (conventional vs. navigation: 1327 ml vs. 925 ml, *p* < 0.001).

Therefore, we conducted this prospective randomized clinical trial to compare the accuracy of the radiographic parameters of the knee implant, total blood loss (TBL), and incidence of thromboembolism between the pinless navigation system (Stryker OrthoMap Express Knee Navigation) and conventional method in patients undergoing MIS-TKA procedures.

## Patient and methods

### Patient enrollment

Between September 2019 and September 2021, a consecutive series of 100 patients underwent unilateral primary MIS-TKA were assessed for their eligibility for inclusion for this study. The inclusion criteria are patients who were between 50 and 90 years of age who had end-stage arthritis of the knee including primary osteoarthritis or secondary arthritis due to inflammatory arthritis, gouty arthritis, or posttraumatic arthritis and underwent unilateral primary MIS-TKA. The exclusion criteria are patients with history of ischemic heart disease, stroke, risk of VTE disease not amenable for tranexamic acid (TXA) administration, preoperative hemoglobin (Hb) level less than 11 g/dl, history of infection or intra-articular fracture of the affective knee, coagulopathy (platelet < 10^5^/mm^3^, prothrombic time, Activated partial thrombin time INR > 1.4), renal function deficiency (Glomerurofiltration rate < 30 ml/min/1.73 m^2^) which is relative contraindicated for chemical VTE prophylaxis, and patients use lifelong anticoagulant therapy, were allergic to TXA or rivaroxaban. The patients were randomly assigned into two groups: navigation group and convention group by an independent research assistant using computer-generated random numbers. The study number according to sequence of the enrolled TKA procedures was marked on the envelopes and the allocation label was concealed by a research assistant not involving in the study. The results of the allocation were decided randomly by the computer-generated method. The surgeon opened the envelopes before preparation of the operation.

### Navigation system

Our navigation system (Orthomap Express, Stryker, Michigan) is an image-free navigation system. No preoperative image is required. The anatomical morphology of knee is registered during operation. This system uses wireless optical tracking technology. Infrared light emitted by diodes placed on navigated surgical instruments is sensed by a camera array (navigation camera) on the computer platform. No need for tracker pins in femur and tibia shafts. The navigation system was only used for distal femur cutting and verification for the resection level in this study. Because the tibia cutting jig of Orthomap Express was much larger than that of MIS instrument, we used the MIS instrument for proximal tibia cut to reduce wound length discrepancy between the two groups.

### Surgical technique

For all arthroplasty, the same cemented prosthesis (NexGen, Legacy, Posterior-Stabilized Prosthesis; Zimmer, Warsaw, Indiana) was used. Almost the total knee surgeries (92/100) were performed by the senior orthopedic surgeon (JWW) with extensive experience in the primary knee replacement using a mini-mid vastus approach under general anesthesia, and other surgeries (8/100) were performed by another orthopedic surgeon (SHY) first, then were finally checked by JWW. A pneumatic tourniquet was inflated to a pressure of 300 mmHg. All arthroplasties are performed in tibia first technique. The proximal tibia cut was done by conventional extramedullary instruments. The femoral preparation technique is depending on which group the patient is assigned in: (1) In the navigation group, the varus/valgus, extension/flexion, thickness of distal cut of femur was determined and done by pinless navigation system. After cutting, the instant information of resection level could be shown on the display screen. If the alignment was satisfying, the anterior/posterior femoral cut, chamfer cut and box resection were done by cutting jig. If a relevant displacement of the cutting jig of at least 1° varus/valgus was measured, correction cutting was performed and the result was digitized again. (2) In the convention of group, the femoral alignment was determined by intramedullary guide. The femoral alignment jig was set to 5°–7° valgus dependent on the preoperative radiograph. A bone plug taken from the resected femoral condyle was sealed into the entry hole of femoral medullary canal before prosthesis placement. A hemovac drain was placed before wound closure, and TXA 1 g was intravenously injected after closure of the joint capsule.

### Postoperative management

Range of motion training by continuous passive motion machine was conducted immediately after operation. The vacuum bag of hemovac drain was not compressed postoperatively for 12 h, then followed by full compression was routinely conducted in all patients. The drain tubes were routinely removed on the second postoperative day (POD). Intravenous prophylactic antibiotic therapy consisting 1 g Cefazolin preoperatively followed by 1 g every 8 h for 3 doses postoperatively was given. Allogenic red blood cell transfusion for 1 unit was given if Hb value was < 8.0 g/dl with clinical manifestation of anemia (dizziness, palpitation, palor, etc.) or Hb value was < 9.0 g/dl in elderly patients with risk of cardio pulmonary complications. Standard venous thromboembolism (VTE) prophylaxis was prescribed in all patients by oral intake of rivaroxaban (Xarelto, Bayer Shering Pharma AG, Wuppertal, Germany) 10 mg-once daily starting on POD 1 for 14 doses. All patients were combined to undergo duplex ultrasound study of both lower limbs to detect the presence of deep-vein thrombosis (DVT) before discharge.

### Outcome assessment

**Fig. 1:**
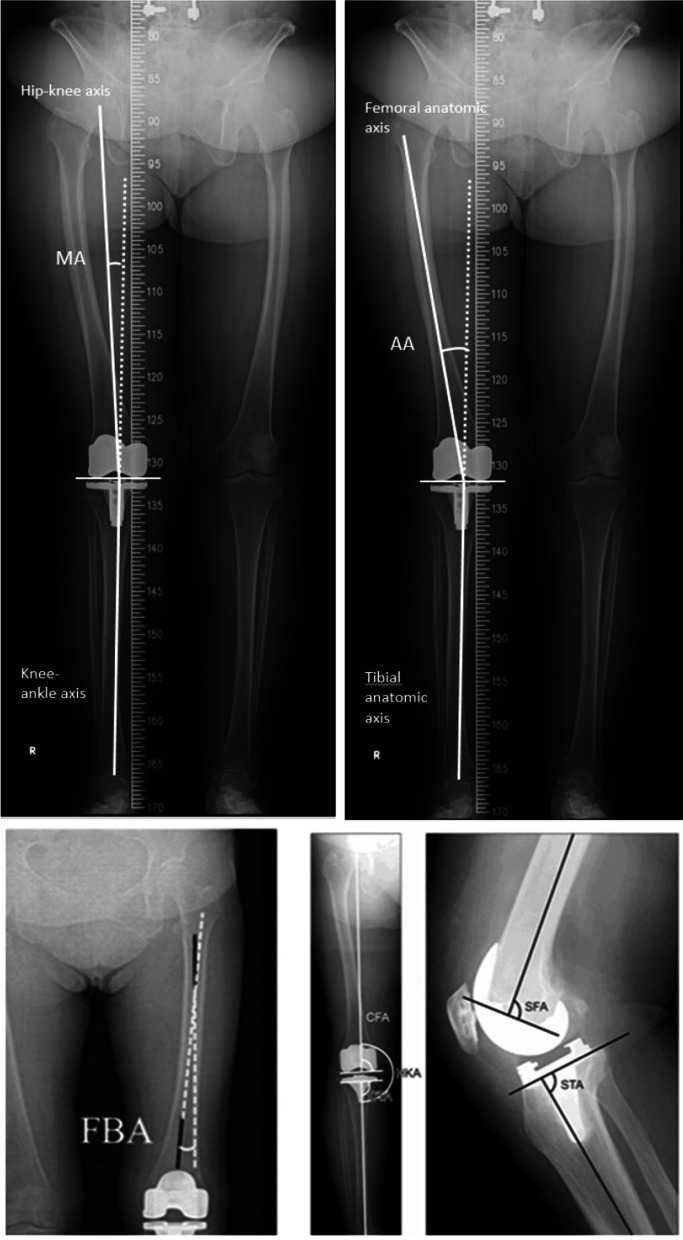
The radiographic parameters of total knee arthroplasty. MA: mechanical alignment (the angle between hip-knee axis and knee-ankle axis on the scenography), AA: anatomical alignment (the ankle between femoral axis and tibial axis on the scanography,), FBA: femoral bowing angle (the ankle between the axis of proximal third femur and the axis distal third femur), CFA: coronal femoral component angle (the angle of femoral component formed with the anatomical axis of femur on coronal view), CTA: coronal tibia-component angle (the angle of femoral component formed with the anatomical axis of tibia), SFA: sagittal femoral component angle (the angle between anatomical axis of the femur and perpendicular line to femoral prosthesis on the lateral view), STA sagittal tibial component angle (the angle between anatomical axis of the femur and perpendicular line to tibial prosthesis)

The primary outcome was the accuracy of the radiographic parameter of the knee implant at 3 months after surgery. The postoperative radiograph was taken after the knee can full extend. The radiographs were performed according to the standard protocol. The radiograph was repeated, if the malrotation of the knee or flexion of knee was seen. The mechanical alignment (the angle between hip-knee axis and knee-ankle axis on the scanography, MA), anatomical alignment (the ankle between femoral axis and tibial axis on the scanography, AA), femoral bowing angle (the ankle between the axis of proximal third femur and the axis distal third femur on the scanography, FBA) were measured on the full-length weight-bearing radiograph according to the method described by Ehrendorfer et al. [19] and Sasaki et al. [20]. The coronal femoral component angle (the angle of femoral component formed with the anatomical axis of femur on coronal view, CFA), coronal tibia-component angle (the angle of femoral component formed with the anatomical axis of tibia on coronal view, CTA), the sagittal femoral component angle (the angle between anatomical axis of the femur and perpendicular line to femoral prosthesis on the lateral view, SFA), sagittal tibial component angle (the angle between anatomical axis of the femur and perpendicular line to tibial prosthesis on the lateral view, STA) in the weight-bearing knee regular radiograph were measured according to the method described by Mizu-uchi et al. [21]. The radiographs were evaluated by using picture archiving and communication systems (PACS) (Fig. 1). All parameters were measured twice by an independent review which was blinded to which group patients were assigned in, and the average of two measurements was taken. The value more than 3° deviation from the standard values of following parameters: MA, CFA, CTA were considered as outliers.

The secondary outcomes included total blood loss (TBL), transfusion rate, surgical wound length, wound complications, and the risk of venous thromboembolism. The TBL was calculated according to Nadler et al. [22], which uses maximum postoperative decrease in the Hb level adjusted for weight and height of the patient. The TBL consists of amount of blood loss calculated from the maximum Hb loss and amount of blood transfused. The event of blood transfusion and calculation of the incidence of transfusion were recorded. The results of duplex ultrasound examinations of both lower limbs were recorded in all patients to see if there was evidence of DVT.

### Statistical analysis

The sample size was calculated based upon our previous study [24], a prospective randomized trial to calculate the perioperative blood loss after total knee arthroplasty. Assuming a mean difference in TBL of 225 mL or greater between the two groups, to obtain a statistical power of 0.90 and an alpha error of 0.05, 30 patients are required in each group. Considering that 10% of patients will be lost to follow-up, and 10% could be expected to have incomplete data, the study aimed to enroll 100 patients.

A paired, two-tailed *t* test was performed to compare differences between the postoperative radiographic parameters between two groups, TBL, surgical wound length, operating time between two groups. Values of *p* ≤ 0.05 were considered statistically significant. Descriptive data, including gender, the incidence of complications, the blood transfusion rate, and deep-vein thrombosis between the two groups were compared using chi-square test or Fisher’s exact test. The intra-rater reliability was measured using one way random single-measure intraclass correlation coefficients (ICCs), and ICC value of more than 0.8 is considered excellent; 0.6–0.8 is good; 0.4–0.6 is moderate and < 0.4 is considered bad correlation. All statistical comparisons were made using the Statistical Package for Social Sciences (SPSS) (version 18; SPSS Inc., Chicago, Illinois).

This prospective randomized clinical trial was registered in the public register at Clinical Trials gov (NCT04235283) and supported by Chang Gung Memorial Hospital (CMRPG8J0311). The protocol was approved by our institutional review board (201900295A3).

## Results

A consecutive series of 100 patients underwent unilateral primary MIS-TKA were included in this study. Seventy-nine patients (79%) were female and 21 patients (21%) were male. The mean age was 69.7 years ranging from 52 to 84 years. The preoperative characteristics of the patients were all similar in the two groups (Table 1).

**Table 1:** Details of the patients

Characteristics	Navigation group (*n* = 50)	Convention group (*n* = 50)	*P* value
Age(yr) (SD;range)	68.78 (6.20;52–81)	70.54 (5.80;60–84)	0.146
BMI (kg/m^2^)	27.00 (4.08;19–36)	27.87 (4.15;20–39)	0.295
BMI (kg/m^2^) > 30	12/50 (24.0%)	13/50 (26.0%)	0.817
Women (%)	39/50 (78.0%)	40/50 (80.0%)	0.806
Side (Right side, %)	32/50 (64.0%)	25/50 (50%)	0.113
History of CAD, H/T, DM	36/50 (72.0%)	36/50 (72.0%)	1.000
ASA-1	0/50 (0.0%)	0/50 (0.0%)	0.130
ASA-2	38/50 (76.0%)	31/50 (62.0%)	
ASA-3	12/50 (24.0%)	19/50 (38.0%)	
PT	10.31 (0.44;9–12)	10.18 (0.36;10–12)	0.104
APTT	26.79 (2.36;19–32)	26.88 (2.21;22–35)	0.855
Preoperative-Hb (g/dl)	13.08 (1.35;10–17)	13.15 (0.92;11–15)	0.774
Platelet count (10,000/L)	235.68 (77.19;107–476)	262.30 (88.44;117–649)	0.112

Continuous data are presented as mean (standard deviation)

*SD* standard deviation, *BMI* Body mass index, *CAD* coronary artery disease, *H/T* hypertension, *DM* diabetes mellitus, *ASA* American Society of Anesthesiologists, *PT* Prothrombin time, *APTT* Activated partial thromboplastin time

Because COVID-19 outbreak occurred in Taiwan in the end of this clinical trial, six patients (three in navigation group and the other three in convention group) did not return our clinics to complete the postoperative radiograph follow-up yet. Total 94 patients have completed the radiographic measures. There were no significant differences in the postoperative MA, AA, and CTA between both groups. The postoperative CFA had significant differences between both groups (89.12 ± 1.83 in the navigation group vs. 90.09° ± 2.18° in the convention group). There were 12 patients in the navigation group (25.5%) and 16 patients in the convention group (34.8%) as the outliers in the postoperative MA. There was no significant difference of the rate of outliers in the postoperative MA (Table 2) between the both groups, as well as the CFA, and the CTA. The excellent intra-observer reliability was confirmed as the ICC values were in the range of 0.87–0.95.

**Table 2:** The postoperative radiographic parameter

	Navigation group (*n* = 47)	Convention group (*n* = 47)	*p* value
Scanograph			
MA (°)	− 0.49 (2.86)	0.35 (3.38)	0.200
MA ≥ ± 3 (no./total no) (%)	12/47 (25.5%)	16/47 (34.8%)	0.367
AA (°)	4.90 (3.58)	6.18 (3.41)	0.081
Knee AP standing view			
FBA (°)	2.60 (3.34)	4.16 (3.90)	0.040
CFA (°)	89.12 (1.83)	90.09 (2.18)	0.022
CFA ≥ ± 3 (no./total no) (%)	6 /47 (12.8%)	10/47 (21.3%)	0.272
CTA (°)	91.01 (1.57)	90.72 (1.96)	0.428
CTA ≥ ± 3 (no./total no) (%)	5/47 (10.6%)	6 /47 (12.8%)	0.748
Knee Lat standing view			
SFA (°)	87.37 (2.61)	86.10 (3.39)	0.045
STA (°)	83.97 (3.01)	82.93 (2.68)	0.080

Continuous data are presented as mean (standard deviation)

In the scanograph, the minus number means the angle of varus deviation and the positive number means the angle of valgus deviation

*MA* mechanical alignment, *AA* anatomical alignement, *FBA* femoral bowing angle in the full-length weight-bearing radiograph, *CFA* femoral component angle, *CTA* coronal tibia-component angle, *SFA* sagittal femoral component angle, *STA* sagittal tibial component angle

The mean TBL in the navigation group was 841 ± 267 mL (198–1431 mL), which was similar to that in the convention group at 860 ± 266 mL (111–1576 mL, *p* = 0.721). There were no differences in the Hb level on POD 1 (11.68 ± 1.15 g/dL vs. 11.46 ± 1.01 g/dL), POD 2 (10.41 ± 1.05 g/dL vs. 10.45 ± 1.11 g/dL) between the navigation group and the convention group, respectively (Table 3). No transfusion event occurred in either group. The length of the wound in navigation group was longer than that in the convention group (9.74 ± 0.94 cm vs. 8.74 ± 0.86 cm, *p* < 0.001). The length of hospital stay was similar in the two groups (Table 3), as were the wound complications up to two weeks after surgery, including ecchymosis, swelling due to hematoma, wound-healing problems and infection. Postoperative DVT was identified by duplex ultrasound in one patient in the navigation group, however, the DVT risk did not differ between the two groups (2% vs. 0%, *p* = 0.315). No medical, wound complications nor death were observed in this study up to 3 months after operation.

**Table 3:** Blood loss and transfusion requirement

Characteristics	Navigation group (*n* = 50)	Convention group (*n* = 50)	*P* value
Wound length in extension (cm)	9.74 (0.94;8–12)	8.74 (0.86;8–11)	< 0.001
Postoperative Hb level day 1 (g/dl)	11.68 (1.15;10–15)	11.46 (1.01;9–14)	0.306
Postoperative Hb level day 2 (g/dl)	10.41 (1.05;9–13)	10.45 (1.11;9–12)	0.847
Postoperative Hb level day 14 (g/dl)	11.13 (1.08;9–14)	11.09 (1.17;9–14)	0.871
Postoperative DVT (no./total no) (%)	1/50 (2.0%)	0/50 (0.0%)	0.315
Blood transfusion (no./total no) (%)	0/50 (0.0%)	0/50 (0.0%)	–
Total blood loss (mL)	841 (267;198–1431)	860 (266;111–1576)	0.721
Length of hospital stay (days)	3.74 (0.69;2–5)	3.72 (0.64;3–5)	0.881

Continuous data are presented as mean (standard deviation)

*Hb* hemoglobin, *DVT* deep-vein thrombosis

## Discussion

In the common practice, the goal of TKA procedure is to restore the MA of the lower limb. Deviation of the MA of the limb away from neutral alignment causes increased force on the concave side of the knee and increases wear rate of the polyethylene insert [3, 4, 23, 24]. The CAS has been used in TKA procedures for decades, numerous studies has demonstrated that the CAS system improves the precision of the prosthesis position and the accuracy of the goal alignment of lower limb [6–10]. In our institute, the previous CAS navigation system requires to set reference arrays with pin fixation of femur and tibia and leading to some pin wound-related complications including bleeding, infection, neurovascular injury and iatrogenic fracture [11–14]. The main advantage of new pinless navigation system (Stryker OrthoMap Express Knee Navigation) is that this system avoids the use of femoral and tibial pins by using mini jigs with trackers that are attached to the joint surfaces, and therefore avoid the pin wound-related complications. However, no checking of the thickness of bone cuts and no guidance to rotation are provided. In a randomized control trial by Harvie et al. [25] it is demonstrated the OrthoMap Express system to be as accurate as previous CAS navigation system (Stryker full navigation system) which femoral and tibial pins were needed. Clement et al. [26] demonstrated that the pinless navigation system significantly reduces the number of outliers for the femoral and tibial components when compared with conventional technique (4% vs. 11%). This study demonstrated that less outliers of MA in the navigation group compared with convention group (25.5% vs. 34.8%). However, there was no statistical significance. All TKA procedures in this study were performed or supervised by an over 35-year experience surgeon (JWW), therefore the accuracy of alignment of TKA may not be so different between TKA whether CAS was used or not. But the CAS system may still be valuable for those patients who has extra-articular deformity of lower limb, or other situations that the intramedullary guide is not available.

TKA using navigation system has some potential benefits from preservation of the medullary cavity of femur, such as less perioperative blood loss [18] and less risk of systemic embolism [27]. In our study, there was no difference in total blood loss or transfusion rate between both groups. It has been well demonstrated in many clinical trials that TXA has the blood-saving efficacy in TKA [28–30]. In the study by Meneghini et al., it is showed that the CAS-TKA has less blood loss (925 ml) than convention group (1327 ml). However, TXA was not used in their study, and the author mentioned that CAS provided blood loss conservation comparable in magnitude to TXA according previous study [31]. In contrast, intravenously injection of TXA 1 g was administrated in all of the patients in our study. The calculated blood loss in TKA in our study was comparable with our previous studies [29, 30]. It indicates that the CAS may not provide more effect in blood loss conservation if the TXA has been used in total knee arthroplasty.

Another advantage of CAS is reducing systemic emboli phenomena in TKA due to avoiding invasion into intramedullary canal [27, 32]. In our study, all patients received chemical prophylaxis of VTE by using rivaroxaban. Only one patient developed DVT detected by duplex ultrasound in lower limb in navigation group, and no symptomatic DVT or pulmonary embolism was noted in our study. The risk of VTE is relatively low under the protection of rivaroxaban, therefore, sample size in this study may not be adequate to show a difference in the risk of VTE.

There were some limitations in our study. First, the preoperative FBA was more significant in convention group (4.1°) than navigation group (2.6°), and the difference may affect the postoperative MA of lower limb. Second, although the pinless navigation CAS has less outliers of MA than convention group, the difference had no statistically significant. A larger number of patients in each arm may be necessary to show a significant difference.

## Conclusion

Our prospective randomized trial demonstrated that the pinless navigation TKA showed a comparable acceptable alignment compared with conventional MIS-TKA. However, the use of the pinless navigation system did not reduce postoperative blood loss nor incidence of DVT as compared with conventional TKA.

## Data Availability

All data supporting our results can be found within the manuscript.
